# Clinical benefits with 300 IR HDM SLIT tablet in Europeans with house dust mite allergic rhinitis: Post hoc analysis of a large phase 3 trial

**DOI:** 10.1016/j.waojou.2023.100849

**Published:** 2023-12-22

**Authors:** Oliver Pfaar, Frédéric De BLAY, Giorgio Walter Canonica, Thomas B. Casale, Philippe Gevaert, Peter W. Hellings, Krzysztof Kowal, Giovanni Passalacqua, Miguel Tortajada-Girbés, Carmen Vidal, Margitta Worm, Farah Bahbah, Pascal Demoly

**Affiliations:** a Department of Otorhinolaryngology, Head and Neck Surgery, Section of Rhinology and Allergy, University Hospital Marburg, Philipps-Universität Marburg, D-35043 Marburg, Germany; b Chest Diseases Department, Federation of Translational Medicine EA 3072, Strasbourg University Hospital, 67000 Strasbourg, France; c Department of Biomedical Sciences, Humanitas University, via Rita Levi Montalcini 4, 20090 Pieve Emanuele, Milan, Italy; d Division of Allergy and Immunology, University of South Florida, Tampa, FL 33612, USA; e Upper Airway Research Laboratory (URL), Department of Otorhinolaryngology, Ghent University, 9000 Ghent, Belgium; f Otorhinolaryngology Department, Otorhinolaryngologist, Head and Neck Surgery, University Hospital of Leuven, University of Leuven, 3000 Leuven, Belgium; g Department of Experimental Allergology and Immunology, Medical University of Bialystok, Sklodowskiej-Curie 24, 15-369 Bialystok, Poland; h Allergy and Respiratory Diseases, IRCCS Policlinico San Martino, University of Genoa, Genoa, Italy; i Department of Pediatrics, Dr. Peset University Hospital, 46017 Valencia, Spain; j Allergy Department and Faculty of Medicine, Complejo Hospitalario Universitario de Santiago, Instituto de Investigación de Santiago (IDIS) and University of Santiago, 15706 Santiago de Compostela, Spain; k Division of Allergy and Immunology, Department of Dermatology and Allergology, Charité-Universitätsmedizin Berlin, 10117 Berlin, Germany; l Independent Researcher, Paris, France; m University Hospital of Montpellier and IDESP, UMR UA11, University of Montpellier - INSERM, Montpellier, France; n Personalized Medicine Asthma & Allergy Unit-IRCCS Humanitas Research Hospital, via Manzoni 56, 20089 Rozzano, Milan, Italy; o Department of Allergology and Internal Medicine, Medical University of Bialystok, Sklodowskiej-Curie 24a, 15-276 Bialystok, Poland

**Keywords:** Allergic rhinitis, Combined score, House dust mite, Quality of life, Sublingual immunotherapy tablet

## Abstract

**Background:**

House dust mite (HDM)-induced allergic rhinitis (AR) is a major cause of allergic respiratory disease. The efficacy and safety of the 300 IR HDM sublingual immunotherapy (SLIT) tablet in patients with moderate-to-severe HDM-AR was confirmed in a large, international, phase 3 randomized controlled trials (RCTs). Here, we analyzed the results in the European population.

**Methods:**

Data from 91 European centers that participated in the international, double-blind, RCT (EudraCT 2014-004223-46, NCT02443805) with the 300 IR HDM SLIT tablet versus placebo over 12 months were analyzed post hoc. The treatment effect in European adults and adolescents was notably assessed through the European Academy of Allergy and Clinical Immunology (EAACI)-recommended combined symptom and medication score (CSMS_0-6_, pre-defined endpoint) and the total combined rhinitis score (TCRS_0-24_, post hoc endpoint, also balanced) during the primary evaluation period (4 weeks at the end of treatment period) using analysis of covariance (ANCOVA).

**Results:**

There were 818 patients who comprised the modified full analysis set in Europe. Over the primary period, the differences in CSMS_0-6_ and TCRS_0-24_ between the 300 IR and placebo groups were statistically significant (p < 0.0001): −0.32 (95%CI [-0.46; −0.17]) and −1.28 (95%CI [-1.63; −0.94]), respectively, with relative differences of −20.9% and −21.2%. All post hoc and the rhinoconjunctivitis quality of life endpoints were significantly improved with 300 IR versus placebo. The 300 IR HDM tablet was generally well tolerated.

**Conclusion:**

This RCT sub-analysis confirmed the 300 IR HDM SLIT tablet is an effective and safe treatment for European adults and adolescents with HDM-AR with clinically meaningful benefits from the patients' perspective.

**Trial registration:**

NCT02443805. Registered on April 29, 2015./EudraCT 2014-004223-46. Registered on September 16, 2015.

## Background

Allergic rhinitis (AR)/rhinoconjunctivitis (ARC) is a disorder of the nose and eyes affecting about one-fifth of the general population primarily driven by an immunoglobulin E (IgE)-mediated type I hypersensitivity response, due to an allergen exposure.^1^^,^^2^ In Europe, the prevalence of diagnosed AR was recently estimated at around 25%.^2^ AR is often characterized by nasal congestion, nasal secretion, post nasal drip, nasal and throat itching, and sneezing, with impact on social activities, social life, school performance, labor productivity, and quality of life, and accompanied by comorbidities such as conjunctivitis or asthma.^2^, ^3^, ^4^, ^5^, ^6^, ^7^ House dust mite (HDM) allergy is a major cause of allergic respiratory disease and a large proportion of patients with AR are sensitized to HDM, predominantly *Dermatophagoides pteronyssinus* and *Dermatophagoides farinae*.^8^, ^9^, ^10^, ^11^

Medical treatment of AR aiming at eliminating both symptoms and inflammatory reactions is mainly based on antihistamines, nasal steroids, or leukotriene receptor antagonists, but symptom relief does not extend beyond the end of treatment.^12^ Allergen immunotherapy (AIT) is currently the only disease-modifying option available for AR, that also controls symptoms, and reduces medication use.^1^^,^^3^^,^^6^^,^^12^, ^13^, ^14^, ^15^ It interferes with the basic pathophysiological mechanisms modulating the allergic immune response and should be used as early as possible in the course of the illness to avoid progression of the disease and new sensitizations.^1^^,^^3^^,^^14^, ^15^, ^16^, ^17^

Various allergen formulations administered either subcutaneously or sublingually (liquid or tablet) have been approved for the treatment of moderate to severe AR with or without controlled allergic asthma induced by HDMs.^12^^,^^18^, ^19^, ^20^, ^21^, ^22^, ^23^ The 300 index of reactivity (IR) sublingual immunotherapy (SLIT) tablet of *D. pteronyssinus* and *D. farinae* extracts (total allergenic activity ratio 1:1) has demonstrated efficacy and safety in adults, adolescents, and children with HDM-induced AR (irrespective of mono- or poly-sensitization status or the concomitant presence of mild asthma) in multinational, double-blind, randomized, controlled trials (RCTs), and has been approved for commercialization in the Asia-Pacific region and Europe.^18^^,^^22^^,^^24^, ^25^, ^26^, ^27^, ^28^ Of these, the most recent, international, phase 3 pivotal RCT confirmed the benefits of a 12-month course of treatment with the 300 IR HDM SLIT tablet versus placebo in a large population of adults and adolescents.^25^ In this trial, the primary endpoint was the average total combined score (TCS, scale 0–15), defined as the sum of the patient's daily rhinitis total symptom score (RTSS, scale 0–12) and daily rescue medication score (RMS, scale 0–3), during 4 weeks at the end of the treatment period. The primary endpoint was met with a difference in total combined score (TCS) of −16.9% between the 300 IR and placebo groups. The pre-specified secondary endpoints consistently confirmed this result demonstrating the robustness of outcomes.

The aim of this post hoc analysis was the evaluation of the European Union (EU) patients that participated in this international trial^25^ to analyze the possible impact of differences in clinical management and/or in patients’ monitoring between regions in addition to possible intrinsic differences between patients from various regions. Moreover, the treatment effect of the 300 IR HDM SLIT tablet in this EU subpopulation primarily assessed on the imbalanced TCS_0-15_ was compared with that on 2 combined scores equally weighing symptom and medication scores as recommended by Health Authorities and international experts:^29^^,^^30^ i) the European Academy of Allergy and Clinical Immunology (EAACI)-proposed total combined symptom and medication score (CSMS_0-6_, a pre-defined endpoint) and ii) the total combined rhinitis score (TCRS_0-24_, a post hoc endpoint). Further insight into how this treatment effect can be translated into a clinically relevant improvement perceivable by the patients is also provided.

## Methods

### Study design and materials

The study design, set up, and results of the large, international, double-blind, placebo-controlled, phase 3 trial (EudraCT 2014-004223- 46 and ClinicalTrials.gov NCT 02443805) have already been published.^25^ Here we report the results observed in the 91 centers from 9 EU countries involved in the trial: Belgium, Bulgaria, Czech Republic, France, Germany, Italy, Poland, Slovakia, and Spain.

Briefly, the screening phase lasted 6 weeks to 6 months (including a 5-week, single-blind, placebo run-in period), the double-blind treatment phase for approximately 12 months, and the follow-up period around 2 weeks. Patients with an average TCS of 5 or more out of 15 over the last 4 weeks of the 5-week run-in period were centrally randomized 1:1 (computer-generated randomization with stratification by study site per participating country) to receive HDM AIT (a sublingual tablet formulation of standardized, purified, freeze-dried, sieved *D. pteronyssinus* and *D. farinae* extracts for daily administration; allergen content per 300 IR formulation: 14–17 μg Der p 1 and 53–68 μg Der f 1; Stallergenes Greer, Antony, France) or placebo. During the dose escalation phase, patients received a 100 IR tablet on Day 1 (under medical supervision for 30 min), 2 100 IR tablets on Day 2, and a 300 IR tablet on Day 3, or matching placebo tablets. The maintenance treatment consisted of 300 IR tablet or placebo once daily.

### Trial population

The main inclusion criteria were reported previously.^25^ Briefly, the population ranged from 12 to 65 years, had physician-diagnosed HDM-induced AR with or without concomitant asthma, and proven sensitization to *D. pteronyssinus* and/or *D. farinae* (skin prick test ≥5 mm than negative control and specific serum IgE ≥3.5 kU/L). The main exclusion criteria followed the guidelines, notably confounding allergies during the 4-week primary evaluation period, partly controlled or uncontrolled asthma according to Global Initiative for Asthma (GINA) 2014 classification,^31^ previous AIT and the standard contraindications to AIT.

### Ethics approval and consent to participate

This double-blind, placebo-controlled, randomized clinical trial was performed in accordance with good clinical practice defined by the International Council for Harmonization and the principles that have their origin in the Declaration of Helsinki and local laws and regulations. All participants or parents or legal representatives (for participants 17 years or younger) gave their written consent to participation, after having been informed of the trial objectives and procedures.

### Endpoints

As previously described, the primary endpoint of the main study was the average of the daily TCS during the 4-week primary evaluation period. The daily TCS_0-15_ was the sum of the patient's daily RTSS_0-12_ and the daily RMS_0-3_. One of the key pre-defined secondary endpoints of this RCT was the CSMS_0-6_ calculated as RTSS_0-12_/4 + RMS_0-3__,_ thus equally weighing the symptom severity and rescue medication use as recommended by the European Medicines Agency (EMA)^30^ and the EAACI.^29^ It is worth noting that the RMS component of both TCS and CSMS used a stepwise approach endorsed by both Health Authorities and international experts, scoring 0 for no treatment, 1 for an oral H1 antihistamine, 2 for intranasal corticosteroid, and 3 for oral corticosteroid.^29^^,^^30^ Based on previously published scores,^32^ an alternate daily medication score (DMS) was analyzed post hoc imputing a score of 4 per tablet of oral antihistamine (maximum daily score = 4), a score of 2 per puff of intranasal corticosteroid (maximum daily score = 8, ie, 2 puffs per nostril) and, if applicable, a score of 12 on the day of oral corticosteroid intake. The DMS ranged from 0 to 12. A second balanced combined score, TCRS calculated as RTSS_0-12_ + DMS_0-12_ and thus ranging from 0 to 24, was further assessed as complementary post hoc efficacy endpoint. Both TCRS_0-24_ and CSMS_0-6_ were analyzed over the primary period in the subgroup of EU patients and compared with the imbalanced TCS_0-15_ and the results in the overall population.

Other clinical endpoints of the main trial were also analyzed post hoc in the EU subpopulation: RTSS, RMS, rhinoconjunctivitis total symptom score (RCTSS), 6 individual rhinoconjunctivitis symptom scores (ISSs), total ocular symptom score (TOSS), rhinoconjunctivitis quality of life questionnaire (RQLQ) score, and safety variables.

### Clinical relevance

Based on the CSMS_0-6_, the treatment effect (ie, the reduction in symptom and medication score with the 300 IR HDM tablet versus placebo over the primary period) was translated into a clinically relevant improvement from the patients' perspective by considering either component of the combined score: RTSS_0-12_ or RMS_0-3_. The reduction in RTSS_0-12_ was correlated to a decrease in symptom severity while the reduction in RMS_0-3_ was correlated to a decrease in the number of days with less therapy for a patient taking antihistamines or nasal corticosteroids daily over the time period.

### Statistical analysis

Data were analyzed with the same methodology used in the main study.^25^

By definition, the modified full analysis set (mFAS) comprised all participants who received at least one dose of the investigated product (IP) and had at least one evaluation of the primary variable during the primary evaluation period.

The baseline period was defined as the last 4 weeks of the 5-week run-in period and the primary evaluation period as the 4 weeks preceding the last record within a time window of last IP ± 28 days for patients treated at least 300 days. The ANCOVA model for appropriate assessment scores (TCS, CSMS, TCRS, RTSS, RMS, RCTSS, ISSs, and TOSS) was executed on the square root of the average score during the primary evaluation period with treatment group as main effect, and the pooled center, square root of the baseline average score, gender, age, asthma and sensitization status as covariates; p-values were two-sided. The back-transformed least squares (LS) means for each treatment group were used to assess the absolute (point estimate) and relative LS mean differences, with the relative LS mean difference (%) = 100 x [(LS mean 300 IR – LS mean placebo)/LS mean placebo].

For the quality-of-life endpoint analysis (RQLQ score), the ANCOVA model did not used the square root transformation.

Additional models for a subgroup analysis of CSMS and TCRS have been performed by adding interaction between the treatment group and the subgroup of patients with “at least one rescue medication use at baseline”.

A Mixed Model with Repeated Measures has been used to assess the treatment effect at study timepoints (3 months, 6 months, 9 months, 12 months) by adding timepoint effect and the interaction between treatment group and timepoint.

The safety analysis was conducted on the safety set comprising all randomized patients having received at least 1 dose of the IP.

Statistical analyses were performed with SAS software (version 9.4, SAS Institute, Inc, Cary, NC).

## Results

### Study patients

Of the 1607 adults and adolescents randomized in this trial, 992 were recruited in European centers and treated either with the 300 IR HDM tablet (N = 498) or with placebo (N = 494). The modified full analyisis set (mFAS) comprised 818 EU patients (384 in the 300 IR group and 434 in the placebo group).

There were no differences between the 300 IR and placebo groups regarding baseline sociodemographic variables, clinical characteristics, and sensitization status of this subpopulation (Table 1).

**Table 1:** Relevant baseline characteristics of the European study population (mFAS).

Characteristic		300 IR HDM (N = 384)	Placebo (N = 434)	Overall (N = 818)
**Gender**n (%)	MaleFemale	211 (54.9)173 (45.1)	229 (52.8)205 (47.2)	440 (53.8)378 (46.2)
**Age classes**n (%)	AdolescentAdult	99 (25.8)285 (74.2)	110 (25.3)324 (74.7)	209 (25.6)609 (74.4)
**Age** years	Mean ± SDMedian	27.7 ± 12.5225.0	26.8 ± 11.4425.0	27.2 ± 11.9625.0
**Duration of HDM allergic rhinitis**years	Mean ± SDMedianRange	8.68 ± 7.7716.130.8–42.3	9.35 ± 8.1396.810.4–47.1	9.03 ± 7.9716.270.4–47.1
**Baseline sensitization status**n (%)	MonosensitizedPolysensitized^a^	242 (63.0)142 (37.0)	268 (61.8)166 (38.2)	510 (62.3)308 (37.7)
**Baseline HDM specific serum IgE levels**^b^ n (%)	≥17.5 kU/L<17.5 kU/L	221 (57.6)163 (42.4)	264 (60.8)170 (39.2)	485 (59.3)333 (40.7)
**Concomitant asthma**n (%)	YesNo	172 (44.8)212 (55.2)	185 (42.6)249 (57.4)	357 (43.6)461 (56.4)

Abbreviations: HDM, house dust mite; IgE, immunoglobulin E; IR, index of reactivity; kU/L, kilounits per liter; mFAS, modified full analysis set; N, number of patients in the analysis set; n, number of patients with data; SD, standard deviation.

a After HDM allergens, the next most frequently observed sensitizing allergens (according to a skin prick test) were grass pollen (in 18.2% of the participants) and cat dander (in 16.5%).

b Baseline HDM specific serum IgE levels: at least one of both (D. pteronyssinus and/or D. farinae) specific serum IgE value(s) ≥ 17.5 kU/L, or both (D. pteronyssinus and D. farinae) specific serum IgE values < 17.5 kU/L

Baseline symptom and medication scores were similar in both treatment groups (Supplemental Table 1) with mean RTSS = 6.82 ± 1.98 (300 IR) and 6.74 ± 1.92 (placebo), mean RMS = 1.01 ± 0.773 (300 IR) and 0.94 ± 0.773 (placebo), mean TCS_0-15_ = 7.83 ± 2.145 (300 IR) and 7.67 ± 2.023 (placebo), mean CSMS_0-6_ = 2.72 ± 0.93 (300 IR) and 2.62 ± 0.88 (placebo), mean DMS = 4.26 ± 3.743 (300 IR) and 3.99 ± 3.755 (placebo), and mean TCRS_0-24_ = 11.07 ± 4.368 (300 IR) and 10.73 ± 4.176 (placebo). The mean overall RQLQ score was 2.40 ± 1.06 in the 300 IR group and 2.47 ± 1.04 in the placebo group.

In the safety set, the median overall exposure to the study treatment was 364 days in the 300 IR and the placebo groups. Compliance was good throughout the treatment period (>90% per group).

### Combined symptom and medication scores: TCS_0-15_, CSMS_0-6_ and TCRS_0-24_

Table 2 shows the results of the 3 combined scores in the overall population and in the EU subpopulation during the primary evaluation period. The LS mean average TCS_0-15_ was significantly lower in the 300 IR group than in the placebo group (point estimates = −0.74, 95%CI [−1.08; −0.38] and −0.90, 95%CI [−1.34; −0.48] in overall and EU evaluable patients, respectively, p < 0.0001 for both), with relative LS mean differences from placebo of −16.9% and −20.3%, respectively. With regard to the balanced symptom and medication scores, average CSMS_0-6_ and TCRS_0-24_, statistically significant LS mean differences between the 300 IR and the placebo groups were also observed. Point estimates were −0.26, 95%CI [−0.38; −0.14] and −1.07, 95%CI [−1.35; −0.79], respectively, in the overall population and −0.32, 95%CI [−0.46; −0.17] and −1.28, 95%CI [−1.63; −0.94], in the European subpopulation (p < 0.0001 for all). The corresponding relative differences of about −18% in all patients and −21% in EU patients were consistent with that of the TCS_0-15_ in each population, respectively.

**Table 2:** Symptom and medication scores (combined or not) during the primary evaluation period (mFAS).

Score	300 IR HDM	Placebo	Difference in LS mean vs placebo			Relative LS mean difference (%)^c^	
	LS mean^a^	LS mean^a^	Point estimate^b^	95%CI	P-value	%	95%CI
**Overall population** (300 IR, N = 586, placebo, N = 676)							
aTCS_0-15_	3.62	4.35	−0.74	[−1.08; −0.38]	<0.0001	−16.9	[−24.0; −9.2]
aCSMS_0-6_	1.19	1.45	−0.26	[−0.38; −0.14]	<0.0001	−18.0	[−25.2; −10.1]
aTCRS_0-24_	4.77	5.85	−1.07	[−1.35; −0.79]	<0.0001	−18.4	[−22.7; −13.9]
**European population** (300 IR, N = 384, placebo, N = 434)							
aTCS_0-15_	3.55	4.45	−0.90	[−1.34; −0.48]	<0.0001	−20.3	[−28.6; −11.3]
aCSMS_0-6_	1.20	1.52	−0.32	[−0.46; −0.17]	<0.0001	−20.9	[−29.4; −11.7]
aTCRS_0-24_	4.78	6.06	−1.28	[−1.63; −0.94]	<0.0001	−21.2	[−26.1; −16.0]
aRTSS_0-12_	3.03	3.82	−0.79	[−1.18; −0.41]	<0.0001	−20.6	[−29.6; −11.3]
aRMS_0-3_	0.24	0.34	−0.10	[−0.17; −0.03]	0.0034	−29.0	[−43.9; −10.9]
aDMS_0-12_	0.95	1.65	−0.41	[−0.56; −0.26]	<0.0001	−30.0	[−38.9; −20.1]

Abbreviations: aCSMS_0-6_, average combined symptom and medication score (scale 0–6); aDMS_0-12_, average daily medication score (scale 0–12); aRMS_0-12_, average rescue medication score (scale 0–3); aRTSS_0-12_, average rhinitis total symptom score (scale 0–12); aTCRS_0-24_, average total combined rhinitis score (scale 0–24); aTCS_0-15_, average total combined score (scale 0–15); HDM, house dust mite; IR, index of reactivity; LS, least squares; mFAS, modified full analysis set; N, number of patients in the analysis set.

a LS mean: Back-transformed LS mean obtained from an analysis of covariance of the square root of the average score during the primary evaluation period.

b Point estimate: LS mean difference between 300 IR and placebo.

c Relative LS mean difference: [(300 IR LS mean – placebo LS mean)/placebo LS mean] x 100

In the EU subpopulation, the components of the combined scores: RTSS_0-12_ and RMS_0-3_ for the TCS_0-15_ and the CSMS_0-6_, RTSS_0-12_ and DMS_0-12_ for the TCRS_0-24_ also showed significant and consistent results (Table 2). Point estimates and relative differences from placebo were −0.79, 95%CI [−1.18; −0.41], p < 0.0001, −20.6% for RTSS_0-12_; −0.10, 95%CI [−0.17; −0.03], p = 0.0034, −29.0% for RMS_0-3_; −0.41, 95%CI [−0.56; −0.26], p < 0.0001, −30.0% for DMS_0-12_.

**Fig. 1:**
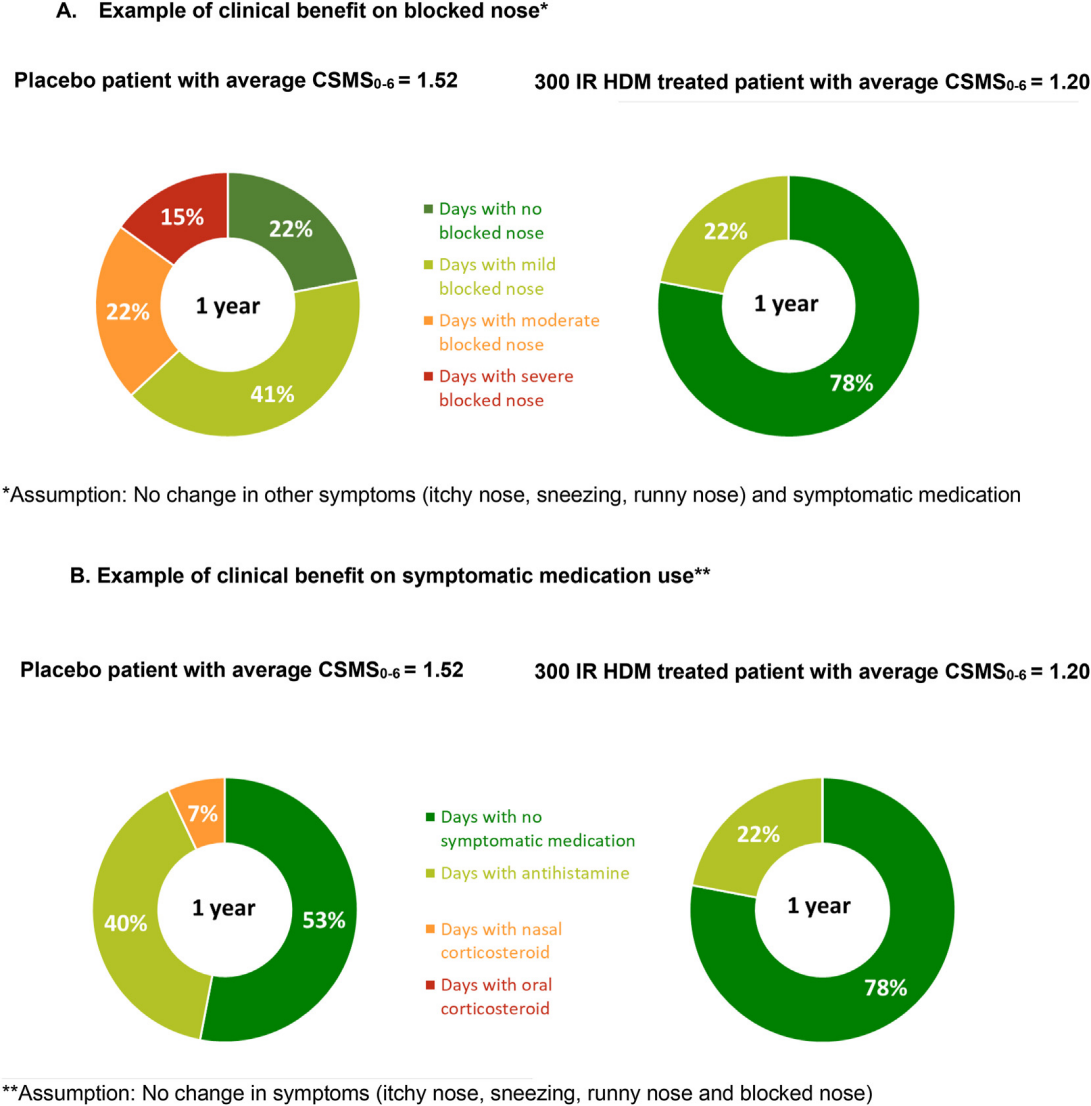
Clinical relevance illustrated with the CSMS_0-6_ difference in European patients (mFAS). CSMS_0-6_, combined symptom and medication score (scale 0–6); HDM, house dust mite; INCS, intranasal corticosteroids; IR, index of reactivity; mFAS, modified full analysis set.

The clinical relevance of the treatment effect of the 300 IR HDM tablet for an EU patient is illustrated in Fig. 1 based on the results of the EAACI-recommended CSMS_0-6_. In calculating the CSMS_0-6_, the RTSS_0-12_ component was divided by 4 to give equal importance to the RMS_0-3_. Thus, proportionately, for each of the 4 rhinitis symptoms initially scored from 0 to 3, a change of one severity level corresponded to a score of 0.25 with a maximum score of 0.75. A reduction of −0.32 in the CSMS_0-6_ may reflect a decrease in one severity class in 1 symptom, for instance blocked nose, for the whole year, assuming the 3 other symptoms remain stable in severity and the use of rescue medication remains also stable. From the score calculation, it is possible to translate the severity of blocked nose into days with mild, moderate or severe symptom or even days with no symptom over the time period. The example in Fig. 1A considers a patient treated with placebo with an average CSMS_0-6_ of 1.52 and suffering from mild blocked nose over 41% of days, moderate blocked nose over 22% of days and severe blocked nose over 15% of days. With a CSMS_0-6_ reduction of −0.32, a patient treated with 300 IR HDM tablet scored 1.20 can expect to be free from moderate to severe blocked nose over 1 year.

Alternately, looking at the RMS_0-3_ component, it is also possible to translate the medication score into days with use of antihistamines, nasal or oral corticosteroids, assuming the 4 rhinitis symptoms remain stable in severity. The example in Fig. 1B considers a patient treated with placebo (CSMS_0-6_ = 1.52) needing oral antihistamines for over 40% of days and nasal corticosteroids for over 7% of days. With a CSMS_0-6_ reduction of −0.32, a patient treated with 300 IR HDM tablet (CSMS_0-6_ = 1.20) can expect to be free from nasal corticosteroids and to reduce oral antihistamine intake over 1 year.

### Additional analyses with CSMS_0-6_ and TCRS_0-24_

In the EU subpopulation, ANCOVA analyses of the average CSMS_0-6_ and TCRS_0-24_ adding interaction between treatment group and the use of any rescue medication at baseline consistently showed that the treatment effect was more pronounced among patients with rescue medication use: CSMS_0-6_ point estimate −0.34, p < 0.0001, relative difference −22.5%; TCRS_0-24_ point estimate −1.38, p < 0.0001, relative difference −22.6%. However, due to the low number of patients who did not use a rescue medication at baseline (n = 43 in 300 IR group, n = 67 in placebo group), the interaction between treatment group and use of rescue medication did not show statistical significance (CSMS_0-6_ and TCRS_0-24_ point estimates −0.14 and −0.62, relative differences −9.7% and −10.9%).

**Fig. 2:**
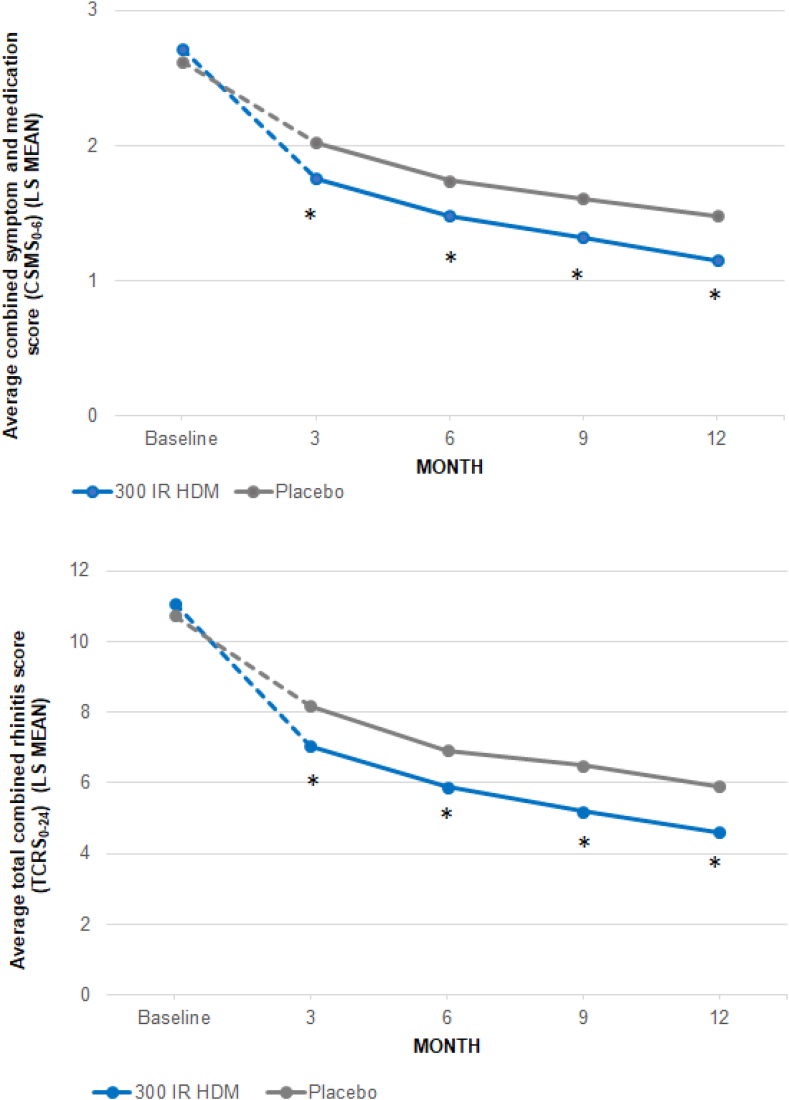
CSMS_0-6_ and TCRS_0-24_ over time in the European study population (mFAS). Average score is mean at baseline, and LS mean at Month 3, 6, 9 and 12 from Mixed Model with Repeated Measures. CSMS_0-6_, combined symptom and medication score (scale 0–6); HDM, house dust mite; IR: index of reactivity; LS: least squares; mFAS: modified full analysis set; TCRS_0-24_, total combined rhinitis score (scale 0–24). Statistically significant intergroup differences ∗p ≤ 0.01.

On the other hand, the treatment effect was assessed over time on CSMS_0-6_ and TCRS_0-24_ using a repeated measures model. A significant difference between the 300 IR and placebo groups was observed from 3 months of treatment onward whichever the score (all p ≤ 0.001, Fig. 2). Relative differences at Month 3, Month 6, Month 9, and Month 12 were −13.6%, −15.0%, −18.1%, −21.9% for the CSMS_0-6_ and −13.8%, −15.0%, −20.0%, −22.1% for the TCRS_0-24_.

### Other endpoints

**Fig. 3:**
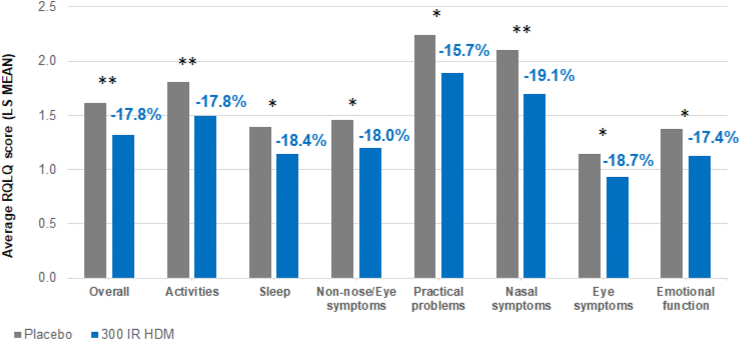
Rhinoconjunctivitis quality of life scores (overall and by domains) at the end of treatment with 300 IR HDM SLIT tablet in the European study population (mFAS). HDM, house dust mite; IR, index of reactivity; mFAS, modified full analysis set; RQLQ, rhinoconjunctivitis quality of life questionnaire; SLIT, sublingual immunotherapy. Statistically significant intergroup differences ∗p < 0.01, ∗∗p ≤ 0.0001.

During the primary evaluation period, all the clinical endpoints assessed in the 10.13039/100006939EU subpopulation collectively demonstrated the 300 IR HDM tablet was significantly better than placebo (Table 3), supporting the results of the combined symptom and medication scores and their components. In addition, the RCTSS, the TOSS, and 5 ISSs: nasal pruritus, sneezing, nasal congestion, ocular pruritus, and tearing improved compared to placebo for 20% or more for each score (Table 3). The 300 IR group and the placebo group differed significantly. With regards to the quality of life, at the end of the treatment period, significant differences were observed in the overall RQLQ score and all the 7 domain scores between the 300 IR and placebo groups (Fig. 3). The point estimate of the overall RQLQ between the 300 IR group and the placebo group at the end of treatment was −0.29 (95%CI [−0.42; −0.15], p < 0.0001) with a relative difference of −17.8%. For the 7 individual domains, the relative differences from placebo ranged from −15.7% for practical problems to −19.1% for nasal symptoms (Fig. 3).

**Table 3:** Other endpoints during the primary evaluation period in the European study population (mFAS).

Score	300 IR HDM (N = 384)	Placebo (N = 434)	Difference in LS mean vs placebo			Relative LS mean difference (%)^c^	
	LS mean^a^	LS mean^a^	Point estimate^b^	95%CI	P-value	%	95%CI
aRCTSS	3.95	4.96	−1.01	[−1.54; −0.50]	0.0001	−20.4	[−29.4; −10.6]
aTOSS	0.71	0.90	−0.19	[−0.34; −0.05]	0.0116	−21.2	[−35.1; −6.1]
average itchy nose score	0.58	0.78	−0.20	[−0.31; −0.10]	<0.0001	−26.2	[−36.9; −14.1]
average sneezing score	0.64	0.84	−0.20	[−0.31; −0.10]	0.0007	−24.3	[−34.7; −13.1]
average runny nose score	0.80	0.98	−0.18	[−0.29; −0.08]	0.0034	−18.8	[−28.4; −8.4]
average blocked nose score	0.80	1.01	−0.21	[−0.32; −0.10]	0.0003	−20.8	[−30.5; −10.1]
average itchy/red eyes score	0.39	0.50	−0.11	[−0.19; −0.03]	0.0078	−22.0	[−35.4; −6.3]
average watery eyes score	0.28	0.36	−0.08	[−0.15; −0.01]	0.0222	−22.5	[−38.2; −4.2]
overall RQLQ score	1.32	1.61	−0.29	[−0.42; −0.15]	<0.0001	−17.8	[−25.1; −10.2]

Abbreviations: aRCTSS, average rhinoconjunctivitis total symptom score; aTOSS, average total ocular symptom score; HDM, house dust mite; IR, index of reactivity; LS, least squares; mFAS, modified full analysis set; N, number of patients in the analysis set; RQLQ, rhinoconjunctivitis quality of life questionnaire.

a LS mean: Back-transformed LS mean obtained from an analysis of covariance of the square root of the average score during the primary evaluation period, except for RQLQ score (no square root).

b Point estimate: LS mean difference between 300 IR and placebo.

c Relative LS mean difference: [(300 IR LS mean – placebo LS mean)/placebo LS mean] x 100.

### Safety

Overall, the safety profile in the EU subpopulation of this RCT was consistent with that in the overall population,^25^ in line with the known safety profile of SLIT. No deaths occurred during the study, and there were no reports of anaphylaxis. No EU participants treated with 300 IR HDM tablet received epinephrine. During the 12-month treatment period, 324 (65.1%) patients in the 300 IR group and 254 (51.4%) patients in the placebo group reported at least one treatment-emergent adverse event (TEAE). The most common TEAEs related to the 300 IR HDM tablet were mild or moderate application-site reactions such as oral pruritus, throat irritation, ear pruritus, and mouth edema. One patient experienced a serious TEAE related to the 300 IR HDM tablet. This was an adult who experienced a severe pharyngeal reaction on the second day of treatment immediately after taking the IP at home. He took cetirizine tablets immediately, and the event resolved 1 h later. The patient discontinued the study on the same day. Forty-one patients (8.2%) in the 300 IR group and 2 patients (0.4%) in the placebo group prematurely discontinued the study due to TEAEs suspected to be drug-related, mostly application-site reactions.

## Discussion

The present study aimed to evaluate post hoc the results of the EU population that participated in the recently published international, randomized, placebo-controlled, phase 3 clinical trial with the 300 IR HDM sublingual tablet,^25^ with a focus on 2 balanced symptom and medication scores following recommendations from Health Authorities and international experts,^29^^,^^30^ the CSMS_0-6_ and TCRS_0-24_.

A large patient population of adults and adolescents with HDM-induced AR was recruited in the primary trial and two thirds (n = 818) of the 1262 patients evaluable for efficacy (ie, included in the mFAS) were European. In this subpopulation as in all patients, treatment with the 300 IR HDM tablet was associated with a significant and consistent reduction in the three assessed combined symptom and medication scores (imbalanced and balanced). Absolute and relative differences from placebo were even greater in EU patients with −0.90 and −20.3%, respectively for the average TCS_0-15_, −0.32 and −20.9% for the average CSMS_0-6_, and −1.28 and −21.2% for the average TCRS_0-24_. Noteworthy, this treatment effect is similar or even better than that observed in a RCT in EU patients with HDM-induced AR with another HDM SLIT tablet (12 SQ-HDM, ALK-Abelló, Hørsholm, Denmark) and using a balanced combined score close to the present TCRS_0-24_.^32^ In that trial, absolute and relative differences in TCRS from placebo were −1.22 and −18%, respectively.

For all 3 combined scores, the relative differences between the 300 IR HDM tablet and placebo in this EU subpopulation were greater than the threshold for difference in the primary outcome relative to placebo recommended by the United States Food and Drug Administration (FDA) to achieve clinically meaningful difference (ie, at least −15% with a 95%CI upper limit of at least −10%).^33^ These differences also exceeded the −20% difference stated by the World Allergy Organization (WAO)^34^ and the Global Allergy and Asthma European network (GA2LEN) of the Allergy and its Impact on Asthma (ARIA) initiative.^35^ However, there is international consensus that a formal validation for the minimal clinically important difference (MCID) of a balanced symptom and medication score as primary endpoint in future trials in AIT is needed for defining the cut-off for a “clinical relevant” treatment effect-size.^29^^,^^36^ From the patient perspective, the results of the EAACI-recommended CSMS_0-6_ supported this clinically relevant effect in that they could be translated into a perceivable reduction in symptom severity, for instance blocked nose (reported as the most bothersome), and/or a decrease or cessation of symptom-relieving medication, notably corticosteroids, over a time period. This is a clinically meaningful result obtained already in the first year of treatment of a perennial disease affecting patients continuously exposed to allergens and having difficulty to control the daily symptoms and reduce their need for rescue medication.

The positive results of the other clinical scores assessing the symptoms and medication use in the EU subpopulation like the components of the combined scores, RTSS, RMS, and DMS as well as the RCTSS and the individual nasal or ocular ISSs, collectively strengthen those of the combined scores, supporting the relevance of the 300 IR HDM tablet. In addition, the overall and the seven RQLQ domain scores were significantly lower in the 300 IR group than in the placebo group. Such an improvement in quality of life, which is known to be greatly impaired in patients with HDM-induced AR, contributes to the meaningfulness of this treatment for the patients.

The 300 IR HDM SLIT tablet is currently marketed in Australia, Japan, New Zealand, South Korea, and more recently Europe. In seeking approval in Europe in 2021, the treatment effect of the product was specifically assessed in the EU population that participated in the primary international trial, which was considered pivotal.^25^ Pivotal trials are normally performed in different countries worldwide to cover different healthcare systems, daily practices, and patient populations. It is remarkable that all the endpoint results in the EU subpopulation consistently exceeded those observed in the overall population.^25^ The lower results in the overall population were essentially driven by differences between active treatment and placebo of lesser extent in other regions and notably in North America. Such a variation between regions may be explained by differences in clinical trial management and/or patients’ monitoring. Indeed, it has been acknowledged that in multi-regional clinical trials, treatment effects of drugs may be impacted by varying factors (including recruitment, compliance, subject retention, medical practice, environmental, cultural, socio-economic factors, etc) across regions or subpopulations.^37^ The EMA ICH guideline E17 on “general principles for planning and design of multi-regional clinical trials” has been recently developed to address these issues but came into effect after the primary international trial was conducted. In addition, differences in patients' clinical characteristics and variations in allergen exposure across regions might have interfered with the trial results.^38^ A post hoc analysis of the patients’ baseline characteristics by region (Supplemental Table 2) revealed that, compared with the EU subpopulation, the North American (NA) was less exposed to HDM, had lower mean levels of HDM-specific IgE, included a higher proportion of polysensitized patients (about two-thirds in North America versus less than 40% in the European Union) with more patients exhibiting confounding sensitizations, therefore more likely to present intercurrent factors that might have affected the efficacy results. In addition, NA participants presented with a longer duration of HDM-AR suggesting they were likely more used to live with their allergy, which could have impacted their way of scoring. Though no notable differences in the combined score results were observed at baseline, the RMS component results were about 2-fold lower in NA versus EU patients and associated with a slightly higher RTSS (Supplemental Table 3). A lower intake of rescue medications in the former might explain the lower decrease in combined score seen in the active group at the end of treatment. Furthermore, the proportion of dropouts was 1.5–1.8-fold higher within the NA versus the EU subpopulation, the primary reason being due to adverse events (AE) in the active group and withdrawal by the subject in the placebo group (Supplemental Table 2). Assuming that the patients who prematurely withdrew due to AEs (mostly reactions to active treatment) were those who might have benefited most from the active treatment if they have continued, this suggests that remaining NA patients were likely less affected so that the difference in scores versus placebo was less marked. Interestingly, the clinical efficacy of the 300 IR HDM tablet was more pronounced in EU patients with severe enough symptoms to require symptom-relieving medication during the baseline period. This reinforces the value of this treatment during the periods with troublesome symptoms, also allowing the patients to reduce their consumption of rescue medications, particularly nasal corticosteroids.

Finally, as expected, the safety profile of the 300 IR HDM tablet in the EU subpopulation was found as acceptable as in the overall population.^25^ No severe anaphylactic reactions were reported.

The strengths of this analysis were the large size of the subpopulation studied consisting of about two-thirds of the primary trial population and the consistency of results whichever the scores evaluated. The main limitation was the treatment duration of 12 months. However, the increased effect of this tablet over the 12-month treatment period, as observed in the overall trial population, is important to highlight given that EAACI guidelines recommend a minimum of 3 years of AIT to achieve long-term efficacy.^1^ Other clinical trials with the 300 IR HDM tablet demonstrated an early onset of efficacy after an interval of only 8–12 weeks, indicating that benefits were apparent also in the first year of treatment.^24^^,^^27^ In addition, one of these trials conducted in Europe showed that efficacy was maintained during a treatment-free follow-up year after 1 year of therapy.^24^ These findings support the potential of AIT for further improvement beyond 12 months and that continuing AIT will ensure to “consolidate” clinically meaningful benefits which can last after treatment cessation.^39^

## Conclusion

The post hoc data from the international, randomized, placebo-controlled clinical trial focusing on the European HDM-AR adults and adolescents confirm the efficacy and safety of the 300 IR HDM SLIT tablet during the first year of treatment. The results were even better than those observed in the overall population. Moreover, when applying the recommended balanced symptom and medication scores CSMS_0-6_ or TCRS_0-24_, a clinically relevant efficacy above the required 20% improvement was shown and translated into clinically meaningful benefits from the patients' perspective. The significant improvement in the overall RQLQ score as well as in all seven domains of the RQLQ unpin the meaningfulness of this treatment for European patients.

## Abbreviations

AIT, Allergen Immunotherapy; ANCOVA, Analysis of Covariance; AR, Allergic Rhinitis; ARC, Allergic Rhinoconjunctivitis; ARIA, Allergy and its Impact on Asthma Initiative; CI, Confidence Interval; CSMS, Combined Symptom and Medication Score; DMS, Daily Medication Score; EAACI, European Academy of Allergy and Clinical Immunology; EMA, European Medicines Agency; FAS, Full analysis set; FDA, Food and Drug Administration; GA2LEN, Global Allergy and Asthma European network; GINA, Global Initiative for Asthma; HDM, House Dust Mite; Ig, Immunoglobulin; IP, Investigational product; IR, Index of Reactivity; ISS, Individual Rhinoconjunctivitis Symptom Score; kU/L, Kilounits per Liter; LS, Least Squares; mFAS, Modified Full Analysis Set; MCID, Minimal Clinically Important Difference; N, n, Number of patients; RCT, Randomized Controlled Trial; RCTSS, Rhinoconjunctivitis Total Symptom Score; RMS, Rescue Medication Score; RQLQ, Rhinoconjunctivitis Quality of Life Questionnaire; RTSS, Rhinitis Total Symptom Score; SLIT, Sublingual immunotherapy; TEAE, Treatment Emergent Adverse Event; TCS, Total Combined Score; TCRS, Total Combined Rhinitis Score; TOSS, Total Ocular Symptom Score; WAO, World Allergy Organization

## Funding

The study as well as the medical writing assistance was sponsored and funded by Stallergenes Greer (Antony, France).

## Availability of data and materials

The datasets used and/or analyzed during the current study are available from the corresponding author on reasonable request.

The data of this post hoc analysis have been presented as abstract at the German Allergy Congress “DEUTSCHER ALLERGIE KONGRESS” (2021), published as “Pfaar O, Kleine Tebbe J, Demoly P, and Bahbah F. Clinical relevance of treatment with 300IR house dust mite SLIT tablet.” Allergo J Int. 2021; 30:215.

## Authors' contributions and consent for publication

All authors participated in the double-blind placebo-controlled clinical trial as country-principal investigators and/or were involved in the discussion of the post hoc analysis, contributed to the manuscript from draft stage and approved the final manuscript for submission.

## Ethics approval and consent to participate

This double-blind, placebo-controlled, randomized clinical trial was performed in accordance with good clinical practice defined by the International Council for Harmonization and the principles that have their origin in the Declaration of Helsinki and local laws and regulations. All participants or parents or legal representatives (for participants 17 years or younger) gave their written consent to participation, after having been informed of the trial objectives and procedures. Data used from RCT (EudraCT 2014-004223-46, NCT02443805).

## Declaration of competing interest

**O. Pfaar** reports personal fees from Stallergenes Greer, during the conduct of the study; grants and/or personal fees from ALK-Abelló, Allergopharma, Stallergenes Greer, HAL Allergy Holding B.V./HAL Allergie GmbH, Bencard Allergie GmbH/Allergy Therapeutics, Lofarma, ASIT Biotech Tools S.A., Laboratorios LETI/LETI Pharma, GlaxoSmithKline, from ROXALL Medizin, Novartis, Sanofi-Aventis and Sanofi-Genzyme, Med Update Europe GmbH, streamedup! GmbH, Pohl-Boskamp, Inmunotek S.L., John Wiley and Sons, AS, Paul-Martini-Stiftung (PMS), Regeneron Pharmaceuticals Inc., RG Aerztefortbildung, Institut für Disease Management, Springer GmbH, AstraZeneca, IQVIA Commercial, Ingress Health, Wort&Bild Verlag, Verlag ME, Procter&Gamble, ALTAMIRA, Meinhardt Congress GmbH, Deutsche Forschungsgemeinschaft, Thieme, Deutsche AllergieLiga e.V., AeDA, Alfried-Krupp Krankenhaus, Red Maple Trials Inc., Königlich Dänisches Generalkonsulat, Medizinische Hochschule Hannover, ECM Expro&Conference Management, Technische Universität Dresden, Lilly, Paul Ehrlich Institut (PEI), Japanese Society of Allergy, Forum für Medizinische Fortbildung (FomF), all outside the submitted work and within the last 36 months; and he is member of EAACI Excom, member of ext. board of directors DGAKI; coordinator, main- or co-author of different position papers and guidelines in rhinology, allergology and allergen-immunotherapy. **F. de Blay** reports financial interests from Stallergenes Greer for participation in advisory boards. **G.W. Canonica** *reports having received research grants as well as being lecturer or having received advisory board fees from:* Allergy Therapeutics, HAL Allergy, Stallergenes-Greer. **T.B. Casale** reports on-financial support from ThermoFisher Scientific, outside the submitted work. **P. Gevaert** reports personal fees and non-financial support from Stallergenes Greer, during the conduct of the study. **P. Hellings** reports grants and personal fees from ALK-Abelló, Astra Zeneca, GlaxoSmithKline, Novartis, Sanofi/Regeneron, Stallergenes Greer and Viatris, outside the submitted work. **K. Kowal** reports personal fees and/or honoraria for lectures, or royalties from ALK-Abelló, AstraZeneca, Aurovitas Pharma, Berlin Chemie, Meda Pharma, Stallergenes Greer and UpToDate, outside the submitted work. **G. Passalacqua** reports consulting fees, honoraria for lectures, and/or research funding from ALK-Abelló, Allergopharma, Lofarma and Stallergenes Greer. **M. Tortajada-Girbés** declares he has no conflicts of interests to disclose regarding this work. **C. Vidal** reports lecture fees from ALK-Abelló, AstraZeneca, Novartis, Sanofi, Leti, Mundipharma, Chiesi and Stallergenes Greer, outside the submitted work. **M. Worm** reports honoraria and/or consultation fees from Abbvie, Aimmune Therapeutics, ALK-Abelló, Amgen, AstraZeneca, Boehringer Ingelheim Pharma, DBV Technologies, GlaxoSmithKline, Kymab Limited, Leo Pharma, Lilly, Mylan, Novartis Pharma, Pfizer Pharma, Regeneron Pharmaceuticals, Sanofi-Aventis, Stallergenes Greer. **F. Bahbah** was a former employee of Stallergenes Greer**. P. Demoly** reports grants from: ALK-Abelló, AstraZeneca, GlaxoSmithKline, Menarini, Puressentiel, Stallergenes Greer, ThermoFisher Scientific, Viatris, Zambon, outside the submitted work.
