# Supplementary material for: Clinical benefits with 300 IR HDM SLIT tablet in Europeans with house dust mite allergic rhinitis: Post hoc analysis of a large phase 3 trial
Source: World Allergy Organ J. 2023 Dec 22;17(1):100849. doi: 10.1016/j.waojou.2023.100849 (PMC10788274; doi:10.1016/j.waojou.2023.100849)
Supplement: Multimedia component 1 [file mmc1.pdf]

Supplemental Table 1. Rhinoconjunctivitis symptom and medication scores (combined or not) at baseline in the European study population (mFAS)

Score		300 IR HDM (N=384)	Placebo (N=434)
**Average RTSS$_{0-12}$**	Mean ± SD	6.82 ± 1.976	6.74 ± 1.919
	95% CI	[6.62; 7.02]	[6.55; 6.92]
	Median	6.44	6.37
	Range	2.6–12.0	2.8–12.0
**Average RMS$_{0-3}$**	Mean ± SD	1.01 ± 0.773	0.94 ± 0.773
	95% CI	[0.93; 1.09]	[0.87; 1.01]
	Median	1.00	0.86
	Range	5.0–24.0	4.4–24.0
**Average TCS$_{0-15}$**	Mean ± SD	7.83 ± 2.145	7.67 ± 2.023
	95% CI	[7.61; 8.04]	[7.48; 7.86]
	Median	7.57	7.26
	Range	3.8–14.4	4.4–14.0
**Average CSMS$_{0-6}$**	Mean ± SD	2.72 ± 0.931	2.62 ± 0.884
	95% CI	[2.62; 2.81]	[2.54; 2.71]
	Median	2.71	2.45
	Range	1.3–5.8	1.1–5.5
**Average DMS$_{0-12}$**	Mean ± SD	4.26 ± 3.743	3.99 ± 3.755
	95% CI	[3.88; 4.63]	[3.64; 4.35]
	Median	3.76	3.25
	Range	0.0–12.0	0.0–12.0
**Average TCRS$_{0-24}$**	Mean ± SD	11.07 ± 4.368	10.73 ± 4.176
	95% CI	[10.64; 11.51]	[10.34; 11.12]
	Median	10.30	9.73
	Range	5.0–24.0	4.4–24.0

**Score**		**300 IR HDM (N=384)**	**Placebo (N=434)**
**RQLQ score**	Mean ± SD	2.40 ± 1.063	2.47 ± 1.039
	95% CI	[2.29; 2.51]	[2.37; 2.57]
	Median	2.34	2.46
	Range	0.1–5.1	0.2–5.6

Abbreviations: $CSMS_{0\text{-}6}$, combined symptom and medication score (scale 0-6); $DMS_{0\text{-}12}$, daily medication score (scale 0-12); HDM, house dust mite; IR, index of reactivity; mFAS, modified full analysis set; N, number of patients in the analysis set; $RMS_{0\text{-}12}$, rescue medication score (scale 0-3); RQLQ, rhinoconjunctivitis quality of life questionnaire; $RTSS_{0\text{-}12}$, rhinitis total symptom score (scale 0-12); SD, standard deviation; $TCRS_{0\text{-}24}$, total combined rhinitis score (scale 0-24); $TCS_{0\text{-}15}$, total combined score (scale 0-15).

Supplemental Table 2. Relevant characteristics in overall study population and in European and North American subpopulations

Baseline characteristic[a]		Overall<br>300 IR (N=586)<br>Placebo (N=676)	EU<br>300 IR (N=384)<br>Placebo (N=434)	NA<br>300 IR (N=164)<br>Placebo (N=203)
**HDM exposure level**	300 IR	163 (35.7)	129 (41.6)	28 (23.0)
**≥2 µg/g dust** n (%)	Placebo	203 (40.3)	151 (45.1)	41 (28.3)
**HDM specific serum IgE**	300 IR	299 (51.0)	221 (57.6)	62 (37.8)
**≥17.5 kU/L**[b] n (%)	Placebo	369 (54.6)	264 (60.8)	79 (38.9)
**D. pte-specific IgE**	300 IR	32.5 (55.83) / 15.3	37.2 (58.11) / 18.3	21.6 (50.16) / 9.5
mean (SD) / median	Placebo	35.8 (67.48) / 16.1	38.6 (73.09) / 18.5	26.5 (50.70) / 11.7
**D. far-specific IgE**	300 IR	35.0 (59.02) / 14.5	41.4 (66.10) / 18.1	20.5 (34.55) / 8.3
mean (SD) / median	Placebo	37.3 (63.45) / 16.8	42.7 (69.53) / 19.6	22.2 (41.33) / 8.8
**Polysensitized** n (%)	300 IR	248 (42.3)	142 (37.0)	102 (62.2)
	Placebo	303 (44.8)	166 (38.2)	134 (66.0)
**With potential confounding**	300 IR	73 (12.5)	25 (6.5)	48 (29.3)
**sensitizations** n (%)	Placebo	76 (11.2)	29 (6.7)	47 (23.2)
**Duration of HDM allergic**	300 IR	11.3 (10.55)	8.7 (7.77)	17.7 (13.53)
**rhinitis** Years, mean (SD)	Placebo	11.3 (9.76)	9.4 (8.14)	15.4 (11.50)

		Overall<br>300 IR (N=802)<br>Placebo (N=805)	EU<br>300 IR (N=498)<br>Placebo (N=495)	NA<br>300 IR (N=256)<br>Placebo (N=259)
**Patients prematurely**	300 IR	238 (29.7)	128 (25.7)	100 (39.1)
**withdrawn**[c] n (%)	Placebo	155 (19.3)	74 (14.9)	69 (26.6)
Primary reason:	300 IR	102 (12.7)	49 (9.8)	50 (19.5)
Adverse event	Placebo	18 (2.2)	9 (1.8)	9 (3.5)
Withdrawal by subject	300 IR	71 (8.9)	39 (7.8)	26 (10.2)
	Placebo	70 (8.7)	28 (5.7)	32 (12.4)

Abbreviations: EU, Europe; HDM, house dust mite; IgE, immunoglobulin E; IR, index of reactivity; kU/L, kilounits per liter; mFAS, modified full analysis set; N, number of patients in the analysis set; n, number of patients with data; NA, North America; SD, standard deviation.

[a]Patient baseline characteristics in mFAS

[b]HDM specific serum IgE levels: at least one of both *D. pteronyssinus* and/or *D. farinae*-specific serum IgE value(s) ≥ 17.5 kU/L

[c]Patients prematurely withdrawn in randomized set

Supplemental Table 3. Rhinoconjunctivitis symptom and medication scores (combined or not) at baseline and during the primary evaluation period in overall study population and in European and North American subpopulations (mFAS)

Score		Overall		EU		NA	
		300 IR HDM (N=586)	Placebo (N=676)	300 IR HDM (N=384)	Placebo (N=434)	300 IR HDM (N=164)	Placebo (N=203)
				**BASELINE**			
**Average $TCS_{0\text{-}15}$**	Mean ± SD	7.87 ± 2.124	7.68 ± 1.940	7.83 ± 2.145	7.67 ± 2.023	7.88 ± 2.080	7.72 ± 1.798
	95% CI	[7.70; 8.05]	[7.54; 8.04]	[7.61; 7.83]	[7.48; 7.86]	[7.56; 8.20]	[7.47; 7.97]
	Median	7.51	7.36	7.57	7.26	7.44	7.48
**Average $CSMS_{0\text{-}6}$**	Mean ± SD	2.62 ± 0.912	2.53 ± 0.847	2.72 ± 0.931	2.62 ± 0.884	2.34 ± 0.799	2.32 ± 0.734
	95% CI	[2.54; 2.69]	[2.46; 2.59]	[2.62; 2.81]	[2.54; 2.71]	[2.22; 2.46]	[2.22; 2.42]
	Median	2.47	2.38	2.71	2.45	2.19	2.13
**Average $TCRS_{0\text{-}24}$**	Mean ± SD	10.70 ± 4.227	10.33 ± 3.949	11.07 ± 4.368	10.73 ± 4.176	9.57 ± 3.567	9.40 ± 3.214
	95% CI	[10.36; 11.04]	[10.03; 10.62]	[10.64; 11.51]	[10.34; 11.12]	[9.02; 10.12]	[8.96; 9.85]
	Median	9.67	9.42	10.30	9.73	8.78	8.61
**Average $RTSS_{0\text{-}12}$**	Mean ± SD	7.01 ± 1.991	6.88 ± 1.870	6.82 ± 1.976	6.74 ± 1.919	7.38 ± 2.001	7.21 ± 1.762
	95% CI	[6.85; 7.17]	[6.74; 7.02]	[6.62; 7.02]	[6.55; 6.92]	[7.08; 7.69]	[6.96; 7.45]
	Median	6.60	6.54	6.44	6.37	7.06	6.93
**Average $RMS_{0\text{-}3}$**	Mean ± SD	0.86 ± 0.772	0.81 ± 0.759	1.01 ± 0.773	0.94 ± 0.773	0.50 ± 0.640	0.52 ± 0.645
	95% CI	[0.80; 0.93]	[0.75; 0.86]	[0.93; 1.09]	[0.87; 1.01]	[0.40; 0.59]	[0.43; 0.61]
	Median	0.71	0.63	1.00	0.86	0.15	0.21

Score		Overall		EU		NA	
		**300 IR HDM (N=586)**	**Placebo (N=676)**	**300 IR HDM (N=384)**	**Placebo (N=434)**	**300 IR HDM (N=164)**	**Placebo (N=203)**
**Average DMS$_{0-12}$**	Mean ± SD	3.69 ± 3.702	3.45 ± 3.634	4.26 ± 3.743	3.99 ± 3.755	2.19 ± 3.096	2.20 ± 2.934
	95% CI	[3.39; 3.99]	[3.17; 3.72]	[3.88; 4.63]	[3.64; 4.35]	[1.71; 2.66]	[1.79; 2.60]
	Median	2.82	2.18	3.76	3.25	0.53	0.89
**PRIMARY EVALUATION PERIOD**							
**Average TCS$_{0-15}$**	Mean ± SD	4.23 ± 2.973	4.84 ± 2.957	4.08 ± 2.881	4.83 ± 2.988	4.63 ± 3.149	4.87 ± 2.903
	95% CI	[3.99; 4.47]	[4.61; 5.06]	[3.79; 4.37]	[4.55; 5.12]	[4.14; 5.11]	[4.47; 5.27]
	Median	4.00	4.76	3.92	4.67	4.46	4.94
**Average CSMS$_{0-6}$**	Mean ± SD	1.42 ± 1.085	1.62 ± 1.079	1.41 ± 1.096	1.65 ± 1.109	1.45 ± 1.058	1.56 ± 1.004
	95% CI	[1.33; 1.51]	[1.54; 1.70]	[1.30; 1.52]	[1.54; 1.75]	[1.29; 1.62]	[1.43; 1.70]
	Median	1.20	1.50	1.17	1.49	1.28	1.54
**Average TCRS$_{0-24}$**	Mean ± SD	5.69 ± 4.546	6.57 ± 4.616	5.61 ± 4.514	6.64 ± 4.716	5.95 ± 4.633	6.36 ± 4.287
	95% CI	[5.33; 6.06]	[6.22; 6.92]	[5.16; 6.07]	[6.19; 7.08]	[5.23; 6.66]	[5.77; 6.95]
	Median	4.76	5.96	4.63	5.94	5.13	6.14
**Average RTSS$_{0-12}$**	Mean ± SD	3.75 ± 2.725	4.29 ± 2.719	3.55 ± 2.588	4.25 ± 2.732	4.24 ± 2.984	4.41 ± 2.719
	95% CI	[3.53; 3.97]	[4.08; 4.49]	[3.29; 3.81]	[3.99; 4.50]	[3.78; 4.70]	[4.03; 4.79]
	Median	3.60	4.12	3.36	4.02	4.13	4.29
**Average RMS$_{0-3}$**	Mean ± SD	0.48 ± 0.693	0.55 ± 0.697	0.52 ± 0.714	0.59 ± 0.728	0.39 ± 0.643	0.46 ± 0.618
	95% CI	[0.43; 0.54]	[0.50; 0.60]	[0.45; 0.60]	[0.52; 0.66]	[0.29; 0.49]	[0.38; 0.55]
	Median	0.05	0.18	0.08	0.22	0.00	0.07

		Overall		EU		NA	
**Score**		**300 IR HDM (N=586)**	**Placebo (N=676)**	**300 IR HDM (N=384)**	**Placebo (N=434)**	**300 IR HDM (N=164)**	**Placebo (N=203)**
**Average $DMS_{0\text{-}12}$**	Mean ± SD	1.95 ± 3.036	2.28 ± 3.153	2.06 ± 3.046	2.39 ± 3.242	1.71 ± 3.066	1.95 ± 2.827
	95% CI	[1.70; 2.19]	[2.04; 2.52]	[1.75; 2.37]	[2.09; 2.70]	[1.24; 2.19]	[1.56; 2.34]
	Median	0.16	0.65	0.33	0.78	0.00	0.29

Abbreviations: $CSMS_{0\text{-}6}$, combined symptom and medication score (scale 0-6); $DMS_{0\text{-}12}$, daily medication score (scale 0-12); EU, Europe; HDM, house dust mite; IR, index of reactivity; mFAS, modified full analysis set; N, number of patients in the analysis set; NA, North America; $RMS_{0\text{-}12}$, rescue medication score (scale 0-3); $RTSS_{0\text{-}12}$, rhinitis total symptom score (scale 0-12); SD, standard deviation; $TCRS_{0\text{-}24}$, total combined rhinitis score (scale 0-24); $TCS_{0\text{-}15}$, total combined score (scale 0-15).
